# Computational identification of adaptive mutants using the VERT system

**DOI:** 10.1186/1754-1611-6-3

**Published:** 2012-04-02

**Authors:** James Winkler, Katy C Kao

**Affiliations:** 1 Department of Chemical Engineering, Texas A&M University, College Station, TX, USA

**Keywords:** Adaptive evolution, hidden Markov Model, Visualizing evolution in real time, Population history

## Abstract

**Background**

Evolutionary dynamics of microbial organisms can now be visualized using the Visualizing Evolution in Real Time (VERT) system, in which several isogenic strains expressing different fluorescent proteins compete during adaptive evolution and are tracked using fluorescent cell sorting to construct a population history over time. Mutations conferring enhanced growth rates can be detected by observing changes in the fluorescent population proportions.

**Results**

Using data obtained from several VERT experiments, we construct a hidden Markov-derived model to detect these adaptive events in VERT experiments without external intervention beyond initial training. Analysis of annotated data revealed that the model achieves consensus with human annotation for 85-93% of the data points when detecting adaptive events. A method to determine the optimal time point to isolate adaptive mutants is also introduced.

**Conclusions**

The developed model offers a new way to monitor adaptive evolution experiments without the need for external intervention, thereby simplifying adaptive evolution efforts relying on population tracking. Future efforts to construct a fully automated system to isolate adaptive mutants may find the algorithm a useful tool.

## Background

Strain development to improve the utility of microbial strains has been a focus of industry for decades. Numerous methods to improve strain characteristics have been developed such as random mutagenesis [1,2], genetic recombination [1,3-5], serial transfers in the presence of various inhibitors [6], and others [7-12]. A novel method to identify the occurrence and expansion of adaptive mutants within an evolving population was recently described by Kao and Sherlock [13], where the population dynamics of strains expressing different fluorescent proteins competing for the limiting carbon source in a chemostat system were monitored using fluorescent activated cell sorting (FACS). This approach (VERT, Visualizing Evolution in Real Time) has been used successfully to elucidate the population dynamics of *Candida albicans *in the presence of an antifungal agent [14] and generate *Escherichia coli *mutants tolerant of n-butanol (Reyes and Kao, manuscript in revision). The use of fluorescent labels improves the ability of the user to track various subpopulations in a quasi-real time fashion compared to microarrays [15] or quantitative PCR [16], and therefore makes the VERT method ideal for identifying adaptive events more quickly than other strain development techniques.

A key aspect of the VERT system and other types of population tracking methods involves analysis of observed population dynamics to accurately detect adaptive events, which are subpopulation expansions triggered by novel adaptive mutants with growth-enhancing mutations. For example, if a growth enhancing mutation (such as one that confers drug resistance or more efficient nutrient uptake) arises in a labeled subpopulation, that specific subpopulation will experience an adaptive event due to an increase in population size. An algorithmic way of analyzing population history data is preferable to human inference, as the former will be more consistent and reliable in most circumstances. A simple yet robust method that can identify adaptive episodes automatically is the hidden Markov model (HMM) [17,18], which involves the computation of the unknown state sequence that is most likely to produce the observed output (emissions) from the process in question. This technique can be applied to determine whether each subpopulation is undergoing an adaptive expansion by examining the visible population proportions, and then computing the probability of an adaptive event based on the model training data. A HMM based approach will also be sufficiently flexible to accommodate variations between experiments arising from species-specific dynamics, data quality issues, and other factors.

In this work, we introduce a population state model (PSM) that employs a hidden Markov model to identify likely adaptive events for several types of chemostat evolution experiments that employed the VERT tracking system. After showing that the PSM predictions are comparable to those obtained from human annotation, properties of several VERT experiments for different species are quantified. Several utilities have also been developed that allow the PSM to quickly analyze raw data and generate predictions concerning experimental evolutionary dynamics. Finally, the ability of the PSM to process other types of evolutionary experiments is discussed.

## Results and discussion

The first step in developing a model to analyze VERT population history is the examination of the population data to develop a method that can determine if the observed population proportion for population *j *at time point *i *represents a statistically significant change compared to point *i-1*. A simple statistical classifier based on data obtained from neutrality (e.g. no adaptive events) experiments is developed to answer this question. This classifier is then utilized to determine emission sequences that represent the statistical significance of population proportion changes for the entire set of VERT data. A hidden Markov-based model, trained with human annotated data, is then applied to determine whether or not a subpopulation is undergoing an adaptive event based on these emissions. Finally, the error rate, behavior, and possible alternative applications of the model are considered.

### Statistical classification of population dynamics data

**Figure 1:**
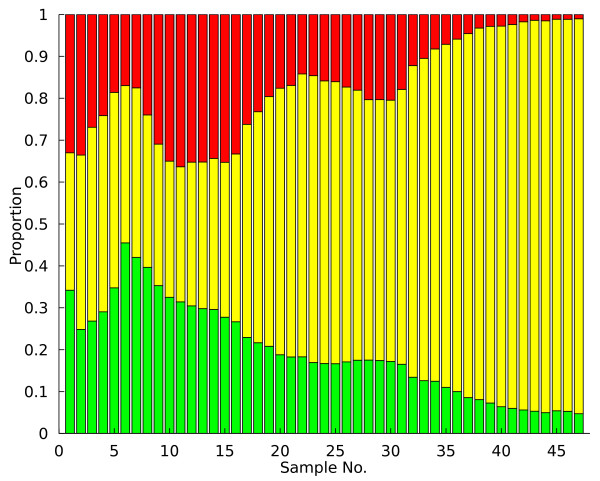
**Data example**. Population dynamics from a yeast population (KK-Large1-2007) selected for growth in glucose limited media.

We seek to analyze the population dynamics that arise during a chemostat evolution experiment. In this type of system, a continuous, constant volume, bioreactor is inoculated with several isogenic microbial populations, each marked with a different fluorescent protein (or equivalent unique label), and evolved for hundreds of generations in the presence of the desired selective pressure. Adaptive mutants from each labeled subpopulation that arise during the course of the evolution experiment trigger an observable increase in the size of the labeled subpopulation, as shown in Figure 1. FACS devices are typically used to track the proportion of each fluorescent strain in the evolving population over time in a series a discrete measurements (typically 1 measurement/day); obtaining continuous data is usually not possible due to experimental and technical limitations. In this case we utilize population dynamics data obtained from evolving yeast and *Escherichia coli *that express several fluorescent proteins.

The population state model utilizes the rate of population expansion for the *j^th ^*subpopulation at time point *i *(r_*pe*,*ij*_) as the measured variable to detect adaptive events from FACS data. Population expansion rate is more practical to work with compared to population proportions over time as adaptive events will change the relative proportions of the subpopulations over time. This property may be calculated directly from FACS data for each time point as follows. First, the proportion of each colored subpopulation *j *of *J *total subpopulations at time *i *(*P_ji_*) is computed from each subpopulation:

(1) $$P_{ji}=\frac{x_{ji}}{x_{j,0}\underset{j\in J}{\sum }{\vec{x}}_{i}}$$

where the summation $\underset{j\in J}{\sum }{\vec{x}}_{i}$ represents the total FACS reading (counts) at the *i^th ^*time point for normalization. This proportion is also divided by x_*j*,0 _to set P_*j*,0 _= 1.0 for all subpopulations, no matter their initial proportion in the inoculum. Since the elapsed time between samples is not necessarily constant over the course of an experiment, let *t_i _*represent the number of generations that have occurred by the *i^th ^*sample. Then, **∀ ***t_i _*>*t*_1_, r_*pe*,*ij*_:

(2) $$r_{pe,ij}=\frac{P_{ji}-P_{j,i-1}}{t_{i}-t_{i-1}}$$

The actual time derivative ${Ṙ}_{j}\left(t\right)$ can used in place of *R_ij _*if continuous measurements are available, as the former contains much more information concerning the process dynamics and will allow more accurate detection of adaptive events.

Estimates for the mean r_*pe*,*ij *_(subsequently *μ_r_*), representing a collection of slope measurements for one subpopulation, and its standard deviation (*σ_r_*) of the same collection for metastable populations are needed to draw inferences about which fluctuations in population proportions are significant. Calibration data in the form of neutrality experiments, where adaptive events are unlikely to occur, can be leveraged to obtain these data. In an ideal case, with a perfectly accurate FACS device and populations with exactly equal fitness, *μ_r _*= *σ_r _*= 0 over the entire dataset; the population proportions would be fixed. In reality, fluctuations affecting both parameters tend to arise due to jackpot mutations, random stochasticity in the populations, or technical issues that generate noise in the data. The neutrality datasets are therefore used to calculate the slope mean and variance. The obtained values for these parameters indicated that *μ_r _*∈ [ - 0.005, 0.004] and *σ_r _*= 0.018 for 64 neutral measurements. The parameter *μ_r _*also serves as an indicator of population stability and is, as expected, indistinguishable from zero at a 95% confidence level.

Generally, *μ_r _*will be approximately zero for fluorophores that have no fitness effect on their host strains. Some fluorescent proteins, such as *tdTomato*, have been observed to decrease strain fitness (data not shown), resulting in negative values of *μ_r_*. The parameter values used here may therefore be unique to specific experimental equipment and fluorophores and should be recomputed for each physically distinct setup.

These properties can be applied to construct a statistical test that will identify when populations begin to expand or contract more rapidly than is expected under the neutral regime. In formal terms, we compare the observed slopes with a random variable R_*pe*,*ij *_drawn from the t-distribution with estimated mean *μ_r _*and standard deviation *σ_r_*. A *t*-test can be used to ascertain whether there is a significant difference between the observed slope and the mean neutral measurement (alternative hypothesis, Equation 4) or if a population is stable (null hypothesis, Equation 3). A Gaussian distribution may also be used in place of the t-distribution if desired; however, if the number of samples is small (less than 30), the t-distribution is more appropriate. The statistic $T=\frac{r_{pe,ij}-\mu_{r}}{\sigma_{r}/\sqrt{n}}$ is used to determine if the difference between the observed and expected slopes is statistically significant.

(3) $$H_{o}:r_{pe,ij}-\mu_{r}=0$$

(4) $$H_{a}:r_{pe,ij}-\mu_{r}\neq 0$$

Each subpopulation of a VERT experiment is analyzed to determine when to reject the null hypothesis in order to classify the data. For slopes that are unlikely to be explained by the null hypothesis (*P *<*α*), the sign of the slope is examined to determine if that point will be identified as a population size increase (positive slope, P) or a contraction (negative slope, N). Slopes that fail to meet the significance threshold, in either direction, are recorded as zero (Z) slopes. The p-value threshold for significance was *α *= 0.10, selected by empirical observation and based on model performance, was used unless otherwise stated. These slope classifications are subsequently used in the population state model described below.

### Definition of the population state model

The basic outline of the population state model (hereafter PSM) exploits the statistical classifier to detect when one subpopulation of labeled cells is undergoing consistent expansion so that the initiation and termination of the expansion can be identified accurately. The mutant is assumed to reach its largest frequency at the latter time point, allowing the experimentalist to more easily isolate the desired mutant from the rest of the population. The model itself utilizes two hidden states: "N" which indicates that a colored subpopulation is not undergoing a population expansion, and "A" to indicate that the subpopulation is experiencing an adaptive event. Annotated training data from 8 multicolored yeast chemostats were used to calculate state transition probabilities within and between the states (*P_AA_*, *P_NN_*, *P_AN_*, *P_NA_*), and the emission probabilities of each symbol (Z, N, and P) in the respective states (*e_A_*(*S*) and *e_N_*(*S*), where *S *∈ {*Z*, *N*, *P*} as defined by the statistical classifier). This process was performed automatically by the model, allowing for the facile incorporation of additional data into the training dataset to improve model accuracy. Training data were used for no other purpose and are not included in any subsequent analyses. Numeric values for each of these parameters are calculated only from the training data and are shown in Table 1. State transition probabilities are adjusted to account for contiguous positive slopes (*C_P_*) or negative and zero slopes (*C*_!*P*_) through the use of an exponentially decay penalty function:

**Table 1:** Population state model parameters

Parameter	Value
State Transition	*P_AN_° *= 0.154, *P_NA_° *= 0.079
Adaptive Emission	*P_N _*= 0.102, *P_Z _*= 0.150, *P_P _*= 0.748
Non-adaptive Emission	*P_N _*= 0.434, *P_Z _*= 0.337, *P_P _*= 0.229

An overview of the Markov parameters used by the population state model. The emission probabilities in the non-adaptive state reflect the symmetry of the slope distribution in the control data and the adaptive emissions are heavily biased towards positive slopes as expected. In addition, the state transition probabilities indicate that entry into and exit from adaptive events is relatively uncommon in the training data.

(5) $$P_{AN}=P_{AN}^{\circ }\left(exp\left(-C_{P}\right)\right)$$

(6) $$P_{NA}=P_{NA}^{\circ }\left(exp\left(-C_{!P}\right)\right)$$

where *P_AN_° *and *P_NA_° *represents that nominal value of each state transition probability. Accordingly, *P_NN _*= 1 - *P_NA _*and *P_AA _*= 1 - *P_AN _*as well. These contiguous counts are reset to zero when symbols outside the considered set (i.e. *Z*, *N *for *C_P_*) are encountered in the data. This modification does represent a divergence from the traditional formulation of a hidden Markov model, where the state at position *i *only depends on position *i*-1. We use this approach to represent the fact that adaptive events, once they occur and survive initial drift, expand in a non-random fashion temporarily. The exponential decay function represents the decreasing probability of transitioning out of an ongoing change in population proportion (i.e. a long adaptive expansion or continual decline); many possible forms for this function exist, but the exponential functions seems to correlate well with the observed population dynamics. This formulation allows for the explicit consideration of the current population state in the chemostat and dramatically improves the accuracy of the model.

**Figure 2:**
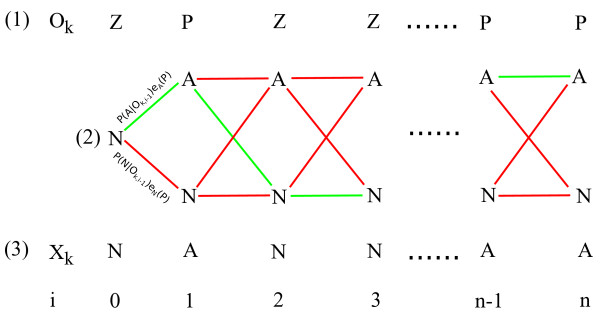
**Markov model decision tree**. Decoding of the hidden Markov states for each labeled subpopulation occurs as follows. (1) the set of emission symbols *O_k _*for a subpopulation is generated from the statistical classifier for all n measurements. (2) The forward Viterbi decoder generates the most likely set of hidden states by choosing the path of maximum likelihood through the system trellis (green lines) based upon the known Markov parameters and *O_k_*. (3) The output set *X_k _*is assembled from these predictions for all observations.

A total of 19 long-term chemostat experiments for *E. coli *(Reyes and Kao, manuscript in revision), *S. cerevisae *[13], and *C. albicans *[14] were analyzed using the PSM. For a given chemostat experiment *k*, the emission sequence *O_kj _*is generated for each of the *j *colored subpopulations using the statistical classifier at significance level *α *= 0.10 (single-tailed). The most likely set of hidden states for the *j^th ^*subpopulation in the *k^th ^*chemostat (*X_kj_*) can then be decoded using the Viterbi algorithm [18] in an iterative fashion:

(7) $$X_{kj}=\left\{argmax\left(P_{ll}\cdot e_{l}\left(O_{k,i}\right),P_{lm}\cdot e_{m}\left(O_{k,i}\right)\right)\forall i\right\}$$

where *l *denotes the previous hidden state and *m *the alternative state (e.g. *A *→ *A or N*). This process is shown graphically in Figure 2. Given that all populations are not expanding immediately after chemostat inoculation, it assumed that all populations are in state N at *i *= 0. In addition, the final adaptive state predictions are translated back one time point (i.e. *i *→ *i *-1) based on empirical observation that doing so improved model accuracy. Model validation was accomplished by comparing the predicted hidden state sequences to human annotation of the 19 chemostats and then computing the number of true positives (A*_mod _*= A*_ann_*), true negatives (N*_mod _*= N*_ann_*), false positives (A*_mod _*= N*_ann_*), and false negatives (N*_mod _*= A*_ann_*) within the computational predictions. Despite the use of true and false designations, the human annotations may not always be accurate representations of the true state of each chemostat population. These error rates can be more accurately interpreted as representing the difference between PSM and human annotations.

The use of a supervised learning approach, though allowing for relatively straightforward development and training of the PSM, does introduce bias into what is considered an adaptive event which in turn affects the model parameters computed from the annotated training set. An alternative approach to HMM training involves the use of unsupervised learning, where the estimated state transition and emission probabilities are computed automatically using algorithms such as Baum-Welch [19]. In essence, this type of HMM training computes the expected number of state transitions and the emission probabilities (in each state) that best fit the provided emission symbols, and then updates the model parameters accordingly. This iterative process continues until the change in HMM performance is below the user threshold. This type of training will be explored in future versions of the population state model.

### Properties of the population state model

Using the procedure outlined previously, the PSM is trained using an annotated dataset from *S. cerevisae *glucose limited chemostats [13]. Depending on the species, length of the evolution experiments, and conditions (mutagenic versus non-mutagenic), it is possible that different estimates of the Markov parameters given in Table 1 may be obtained depending on the dataset used for model training; however, the calculated probabilities seem reasonable in light of the experimental population dynamics. Non-adaptive events typically have slopes that are close to zero (*P *> 0.10) with the remaining events split evenly between positive and negative slopes (*P *< 0.10). Adaptive events are predominately weighted towards producing measurements with positive slopes as is trivially expected. The behavior of the PSM is overall most affected by the state transition properties *P_AN_° *and *P_NA_° *as these parameters control how quickly the model responds to changes in chemostat dynamics.

In order to quantify the error rate of the model more precisely, the PSM was used to generate hidden state predictions for a collection of chemostat evolution experiments for *E. coli*, *S. cerevisae*, and *Candida albicans *which were then compared to human annotations. As can be seen in the error rates reported in Table 2, the model achieves a prediction accuracy rate of 85% to 93% for the examined data. Discrepancies between the model and the annotated states typically arise from the inability of the statistical classifier to call positive slopes that do not meet the statistical threshold for significance; slow adaptive events (subpopulation growth rate < 0.0025 gen^1 ^at *α *= 0.10) may therefore be missed by the model. While these events are relatively rare and therefore do not impact the accuracy of the PSM substantially, slow adaptive events may harbor new lineages or additional mutations that can shed light on the condition being evaluated. However, even in light of this deficiency, the chemostat properties in Table 3 calculated using the PSM are not significantly different from those obtained from human annotation. In addition to these continuous culture systems, the PSM was also able to accurately annotate VERT data obtained during a batch serial transfer experiment (data not shown).

**Table 2:** Population state model error analysis

System	Description	TP_*A *= *A*_	TN_*N *= *N*_	FP_*A *= *N*_	FN_*N *= *A*_
*C. albicans*	Fluconazole challenge	0.213	0.598	0.108	0.082
*E. coli*	Butanol challenge	0.167	0.683	0.043	0.108
*S. cerevisae*	Glucose limitation	0.216	0.720	0.044	0.020

Error rates are calculated by comparing the set of hidden states generated by the PSM to human annotation and then applying the translation method discussed in the model description. Model parameters were calculated with *α *= 0.10 for the statistical classifier. The preponderance of slow adaptive events in the *E. coli *chemostats accounts for the increased proportion of false negatives generated by the model. Overall, the PSM predictions agree quite well with the annotated data.

**Table 3:** Analysis of population dynamics

System	AE/gen-color	Rate of PEX†	AE Length (*s*)
**Human Annotation**			
*C. albicans*	0.015 (0.007)	0.0058 (0.016)	3.26 (2.12)
*E. coli*	0.017 (0.005)	0.0065 (0.009)	1.80 (0.96)
*S. cerevisae*	0.008 (0.005)	0.005 (0.005)	4.124 (3.47)
**Model predictions**			
*C. albicans*	0.016 (0.009)	0.010 (0.015)	3.83 (2.79)
*E. coli*	0.013 (0.010)	0.005 (0.004)	2.46 (1.62)
*S. cerevisae*	0.009 (0.005)	0.005 (0.005)	4.33 (3.43)

Properties of adaptive events (AE) are calculated from the human annotated data and the predictions of the PSM to highlight differences between the annotation methods. The average value and (standard deviation) are provided for each parameter of interest. There are not statistically significant differences between each type of chemostat (i.e. *E. coli *versus yeast) at the *α *= 0.05 level. †PEX: *p*opulation *ex*pansion, defined as *ΔP_ij_/Δt *(*t*: generations).

### Example application: analysis of a yeast chemostat

**Figure 3:**
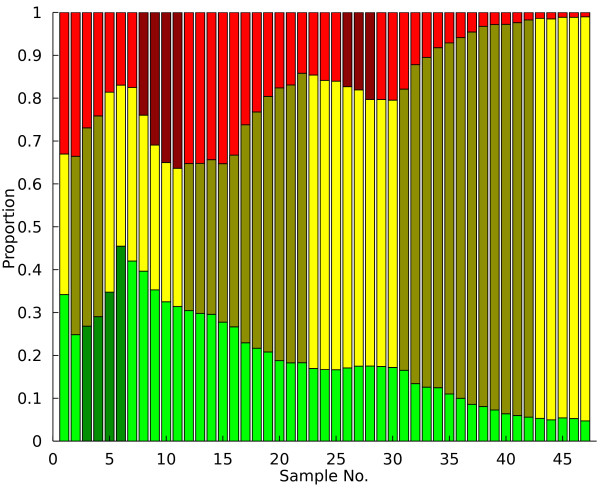
**Output Example**. Using the experimental dynamics in 1 and the PSM, the timing of each adaptive event in the chemostat is calculated and displayed for the user as shaded time points.

An example of the PSM predictions is shown for a yeast chemostat (Large1-KK-2007) in Figure 1. In this system, three fluorescent strains are competing for access to limited glucose; adaptive events occur as individual acquire mutations that affect the rate of glucose transport into the cell. Upon visual inspection of the raw population data in Figure 1, an experienced VERT user would likely conclude that adaptive events (expansions) occur several times in each subpopulation and that the mutations conferring the greatest fitness advantage occur in the yellow population. Analyzing these population dynamics using the PSM produces the adaptive event predictions shown in Figure 3 as shaded regions within each subpopulation. While the model is very successful at identifying the adaptive expansion regions that would likely be identified during a qualitative analysis in this case, it should be noted that excessive noise in the raw FACS data arising from experimental error or constantly varying selective pressure may render adaptive event identification more error prone. However, this tendency should not be a problem in most situations.

**Figure 4:**
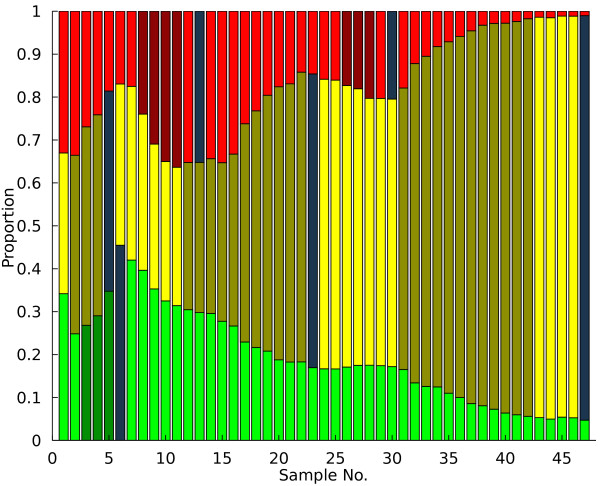
**Sampling Example**. Following the identification of adaptive events, estimates of optimal sampling points as described in the text are then computed to further assist in mutant isolation.

Now that adaptive events have been identified, adaptive mutants must be isolated from the chemostat population. Preserved population samples stored at -80°C may be regrown in the selective media, plated, and analyzed to determine which clonal isolate contains the adaptive mutation. Since any sample can potentially contain the mutant of interest, an additional tool based on the emission sequence generated by the statistical classifier and the hidden state data from the PSM was developed to guide sampling efforts so that the sample with the highest proportion of the adaptive mutant is identified. Firstly, the endpoints of each contiguous series of adaptive events ("A" states) are identified using the PSM output. Then, for each distinct adaptive event the emission sequence for that subpopulation is examined until a "N" symbol (statistically significant negative slope) is found at point *i*. The sampling suggestion is then set to *i-1 *as that time point likely contains the largest proportion of the mutant. Applying this procedure to this chemostat yields the sampling predictions highlighted in dark blue in Figure 4. The identified sampling points are either immediately adjacent to each adaptive expansion (if followed shortly by another expansion in a different subpopulation) or in the case of the final, high fitness yellow mutant, some distance away from the calculated adaptive event endpoint. The latter estimate arises from the fact that the yellow subpopulation essentially overran the chemostat environment, so that the optimum sampling point coincided with the final population measurement. Quantitative PCR measurement of allele frequency in each population supports this sampling scheme [13]. Altogether, these sampling suggestions provide a useful and accurate tool for the experimentalist to optimize their VERT experiment and minimize unnecessary mutant isolation.

### Distribution of adaptive events

**Figure 5:**
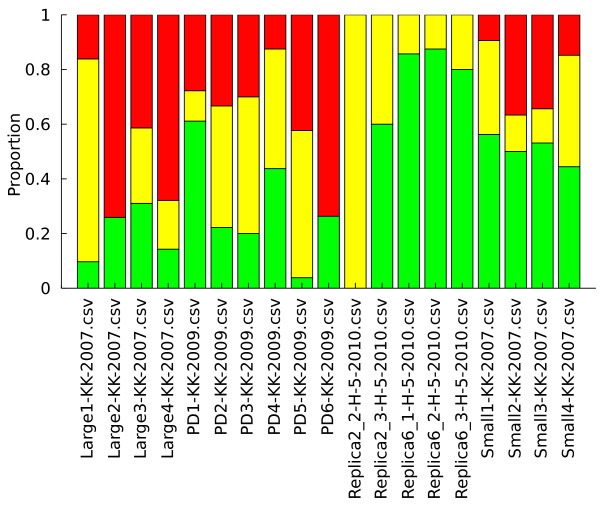
**Distribution of experimental adaptive events**. The relative proportions of adaptive events in each subpopulation, calculated using the PSM, in the three chemostat systems considered here. The neutrality of the fluorescent proteins implies that there should not be a consistent bias of adaptive events towards any particular color, and this assumption holds here for all chemostats. Statistically significant differences in adaptive event abundance between the labeled populations would imply the presence of jackpot mutants.

In addition to the adaptive events themselves, how these events are distributed between the various evolving subpopulations is also of interest to detect differences in the initial seed populations or fitness effects of the fluorescent labels. If one label has a significant detrimental impact upon strain fitness, it is unlikely many detectable adaptive events will occur in that particular subpopulation. The PSM was utilized to calculate the number of adaptive events, weighted by length, per subpopulation for the entire set of available data (Figure 5). A consistent bias towards adaptive events in a particular subpopulation for chemostats seeded from the same initial inoculum may indicate the presence of a beneficial mutant that arose prior to exposure to the selective pressure in question (a jackpot). A statistical method for identifying this type of biased population dynamics will be developed to investigate this phenomenon in a rigorous manner.

#### Application to other evolution systems

Despite the usage of the VERT system and data in developing the PSM, there is no explicit dependence of the PSM on VERT data. Any method that can generate similar population histories over time (e.g. microarray or qPCR methods) can also be integrated into the PSM. The only requirement is that comparable neutrality experiments and annotated experimental data must be generated using the proposed alternative so that the PSM can estimate the required HMM parameters. The current implementation of the PSM will automatically calculate all of the necessary parameters except for *μ_r _*and *σ_r _*for the new type of measurements, both of which must be determined by the end-user as described previously. After this calibration procedure, the PSMshould be able to analyze population histories obtained from alternative methods.

Another potential application of the PSM is the construction of a mostly automated system (e.g. *autoVERT*) for the observation and isolation of adaptive mutants. Unlike serial transfer (batch) evolution system that require periodic transfers of culture to fresh medium, the continuous culture system used to generate the VERT population histories can be adapted to minimize required external intervention to adjust the nominal media composition. The second part of an automated system is identifying when adaptive events occur so that samples of the population can be saved (on solid media or as frozen stocks) for later manual analysis. Given that the PSM has been shown to be effective in accomplishing this task, it may be possible to adapt this model to construct such a system. Additional work is needed to optimize the PSM for this type of data forecasting as the model was primarily constructed for retrospective analysis of VERT experiments.

## Conclusions

The population state model offers the ability to automatically detect adaptive events within fluorescent microbial populations easily and without the need for user intervention. A variety of VERT experimental properties may also be determined, enabling a quantitative comparison between the evolutionary dynamics of different VERT experiments involving various inhibitors or species of interest. Comparison to human analysis of VERT experiments revealed that the PSM produced highly accurate predictions for adaptive events and sampling time points. This algorithm represents an important new tool for the analysis of population dynamics over time and will be integral in any VERT system capable of automatic identification of adaptive mutants.

## Methods

### Experimental procedures

The specific experimental procedures for the VERT experiments used in this study are detailed elsewhere [13,14]. The first requirement is that strains with chromosomally integrated fluorescent proteins (*e.g*. RFP, GFP, YFP) be constructed. The labeled strains must then be assayed to ensure fluorescent protein expression has a neutral effect on strain growth rates. Once label neutrality has been established, equal proportions of each strain are inoculated into a continuous culture system (chemostats) or batch flasks and sampled daily using a FACS machine to determine the size of each labeled subpopulation. The complete series of FACS measurements for a VERT experiment (see Figure 1) can be interpreted as a quantitative measurement of population dynamics. These data form the basis of the population state model developed in this work.

### Computational procedures

All software was implemented in MATLAB R2010a without additional toolboxes on Mac OS × 10.6. Data for model training were annotated and stored as comma separated value files (see Additional File 1). Experimental data was also stored in a similar format without annotations. The purpose of each program used in this work is described in Table 4.

**Table 4:** Description of PSM submodules

File	Purpose
*driverVERT*	Generates data, tables, figures for this work
*errorRates*	Compares state annotations to state predictions
*sampleGuider*	Optimal sampling predictions
*statClassifier*	Converts FACS data to emission sequences
*statisticsVERT*	Analyzes statistics of interest (e.g. AE/gen-color)
*vertDistribution*	Generates distribution of adaptive events for a dataset
*vertHMM*	Converts emission sequences to state predictions

## Competing interests

The authors declare that they have no competing interests.

## Authors' contributions

JW proposed the concept, annotated the data, constructed the model, analyzed the experiments, and wrote the paper; KCK generated the *Candida *chemostat data, oversaw the project, and wrote the paper. Both authors read and approved the final manuscript.

## Supplementary Material

Additional file 1 **Population State Model (JBE V1)**.zip. The collection of MATLAB and data files necessary to use the PSM and generate the figures, data presented in this work. Click here for file
