# Supplementary figures and images for: Computational identification of adaptive mutants using the VERT system
Source: J Biol Eng. 2012 Apr 2;6:3. doi: 10.1186/1754-1611-6-3 (PMC3351376; doi:10.1186/1754-1611-6-3)

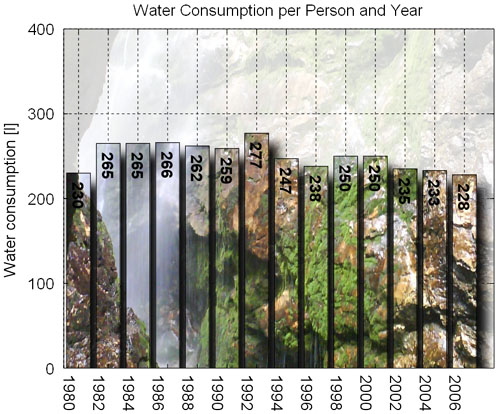

Supplement: Additional file 1 — Population State Model (JBE V1).zip. The collection of MATLAB and data files necessary to use the PSM and generate the figures, data presented in this work. [file 1754-1611-6-3-S1.ZIP › chemostat_data/plot2svg/demo_svg_water.jpg]
